# Clinical and epidemiological characteristics of amyotrophic lateral sclerosis in an Egyptian cohort

**DOI:** 10.1007/s10072-024-07760-w

**Published:** 2024-09-30

**Authors:** Radwa Soliman, Enass Onbool, Kareem Omran, Nagia Fahmy

**Affiliations:** Neuromuscular Unit, Neurology and Psychiatry Department, Faculty of Medicine Ain Shams University, Cairo, 11566 Egypt

**Keywords:** Amyotrophic lateral sclerosis, Epidemiology, Registry, Clinical, Disease progression, Egypt

## Abstract

**Objective:**

Amyotrophic lateral sclerosis (ALS) is a fatal neurodegenerative disorder associated with progressive loss of motor neurons. It is a growing and underestimated disease, prompting this epidemiological study to describe the characteristics of ALS in Egyptian patients.

**Methods:**

This is a prospective hospital based study. ALS patients were recruited consecutively from Neuromuscular Unit in Ain Shams university Hospital from December 2018 to June 2023. Demographic data and disease related parameters were recorded.

**Results:**

203 ALS patients had a mean age of onset equal 39 years and an inter quartile range IQR of (28.00–51.00). 76% of the cases were spinal onset ALS. Median disease duration was 2 years with IQR of (1–4 years); male to female ratio was 2.5:1; 18% of patients were familial ALS (FALS), while 19% were Juvenile ALS (JALS). Median diagnostic delay was 12 ± (6–36) months. Median Amyotrophic Lateral Sclerosis Functional Rating Scale Revised scores (ALSFRS-R) at presentation was 34.5 IQR of (26.00–40.00). Also, the mean rate of disease progression ALSFRS-R decline [points/month] was 0.76 ± 0.51.

**Conclusion:**

Our cohort was characterized by a younger age of onset, male predominance, more familial cases, within average Initial ALSFRS-R scores as well as diagnostic delay. Juvenile ALS patients were much more common in our population. These findings suggest an influential presence of genetic and epigenetic factors affecting the clinical phenotype of Egyptian ALS patients.

## Introduction

Amyotrophic lateral sclerosis (ALS) is the most frequent of the motor neuron diseases (MND); it's a progressive neurodegenerative disease that affects nerve cells in the brain and the spinal cord, causing the loss of motor function. Disease progression ultimately leads to respiratory failure, which is a leading cause of death in ALS [1].

The incidence of ALS was shown to vary between 0.6 and 3.8 per 100,000, while the prevalence varies between 4.1 and 8.4/100,000 [1–4].

The etiology of ALS is complex and still poorly understood [5], and probably combines multiple genetic and environmental factors [6].

Most ALS cases (around 90%) are believed to occur sporadically (sporadic ALS [sALS]) while nearly 10% report known family history of the disease (familial ALS [fALS]) [7].

Motor neuron degeneration causes progressive weakness of limb, thoracic, abdominal, and bulbar muscles; thus leading to a heterogeneous clinical spectrum that encompasses several phenotypes; including varying age and sites of onset, rates of progression of motor symptoms, as well as an occurrence of cognitive impairment and other extra motor manifestations [8].

It is a fatal disease where patients usually die within 3 to 5 years from onset; however, studies show wide variability in disease progression [9]. This could be attributed to the heavy influence of genetics, environmental factors and ethnicity on the characteristics of ALS, thus it may vary across populations [9, 10], however, little is known about its incidence and clinical features in certain regions, including Egypt where there is scarcity of information [11].

In fact, there are very limited studies that described ALS among African populations [12].

Thus, more studies are needed in this region. That's why we conducted our study to describe the epidemiologic and clinical data of ALS in Egypt.

## Methods and patients

This is a prospective hospital-based study conducted in neuromuscular unit in Ain Shams University Hospital; a tertiary care centre for ALS, receiving patients from all over Egypt. Patients were recruited consecutively between December 2018 and June 2023. All ALS patients included were diagnosed by senior experts according to the El Escorial revised criteria [13].

Patients with diagnoses of other neurodegenerative or neuromuscular diseases were excluded. demographic and clinical data were recorded, including age of disease onset in order to stratify patients into age groups and study the differences with juvenile onset (JALS) who are typically defined as patients with onset before the age of 25 years [14] ‘Young-onset’ amyotrophic lateral sclerosis is considered to be similar to ‘classic’ ALS with mixed upper and lower motor neuron features commencing before an arbitrary cut-off age of 45 years and apparently sporadic [15]; family history to identify fALS patients [7]; residency which was defined as a place where a patient had lived for > 10 years [16]; occupation, medical conditions; site of onset (bulbar or spinal); El Escorial classification at diagnosis, diagnostic delay (duration from onset to diagnosis); Arabic version of Amyotrophic Lateral Sclerosis Functional Rating Scale Revised (ALSFRS-R) to asses severity [17] score at diagnosis and follow-up months; stage of the disease kings scale [18]; cognitive functions were assessed via the Egyptian version of the Edinburgh Cognitive And Behavioral Amyotrophic Lateral Sclerosis Screen (ECAS*‑EG*) [19]. We also screened patients for the presence of other extra motor affection (extrapyramidal, cerebellar, sensory disturbances, dysautonomia). The rate of disease progression (measured by the assessment of ALSFRS-R score at the time of diagnosis then sequentially in the follow-ups) was then calculated as the mean points decreased from the time of diagnosis to follow-up, calculated in months. The monthly ALSFRS-R progression rate was calculated using the delta formula: (ALSFRS-R at diagnosis–ALSFRS-R at follow-up)/ (last date–first date) [20].

## Statistical methods

Data analysis was done using SPSS 27 (IBM SPSS Statistics for Windows, Version 27.0. Armonk, NY: IBM Corp). Quantitative data were presented using minimum, maximum, means and standard deviations. For non- parametric data, medians and IQR were used.

Qualitative data were presented using count and percentage.

Student t test was used to compare quantitative data between two independent groups (and Mann Whitney test as a non-parametric test).One way ANOVA test was used to compare quantitative data between more than two independent groups (Kruskal Wallis test as a non-parametric test). Pearson and Spearman’s correlation tests were used to measure correlation between different quantitative data.

Chi square and Fisher exact tests were used to compare qualitative data between different groups. P value less than or equal to 0.05 was considered statistically significant.

## Results

### Phenotype

A total of 203 ALS patients were recruited consecutively in the period between December 1st, 2018, and June 1st, 2023.

Demographic and clinical data are shown in Table 1. There was 146 male and 57 female with M: F ratio equal 2.5:1. Median age for the patients at presentation was 42 and IQR of (34.00–54.00); over 56% were living in Cairo. 76. 3% had spinal onset (155 cases) whereas bulbar onset was seen in 23.6% of patients (48 cases). Consanguinity was positive in 34% of cases, 18% of patients were familial cases. Median disease duration was 2 years with IQR of (1–4 years). 64% of patients received Riluzole 100 mg daily while 20% received both Riluzole and Edaravone.

**Table 1:** Demographic and clinical data of Egyptian ALS patients

	Min	Max	Median	IQR
Age (years)	11.00	70.00	42.00	34.00–54.00
age of onset	3.00	69.00	39.00	28.00–51.00
duration (years)	0.25	20.00	2.00	1.00–4.00
time from onset to diagnosis (months)	2.00	120.00	12.00	6.00–36.00
ALS-FRS R	7.00	48.00	34.50	26.00–40.00
ECAS*-EG*	40	130	98.00	70.00–108.00
			*N*	%
Sex	Male		146	71.9%
	Female		57	28.1%
Marital status	Married		137	67.8%
	Single		65	32.2%
Residency	Cairo		110	56.4%
	Outside Cairo		85	43.6%
Occupation	Manual worker		73	36.5%
	Office work		67	33.5%
	Housewife		31	15.5%
	Student		15	7.5%
	Not working		14	7.0%
Consanguinity	No		133	65.6%
	Yes		70	34.4%
Type S/F	Sporadic		167	82.3%
	Familial		36	17.7%
El Escorial classification at diagnosis	Definite		162	79.8%
	Probable		28	13.7%
	Probable laboratory supported		8	3.9%
	Possible		5	2.4%
Extra motor affection at presentation	No		69	57.0%
	Yes		52	43.0%
Need for nasogastric tube at presentation	No		149	80.5%
	Yes		36	19.5%
Need for ventilation support at presentation	No		151	81.6%
	Yes		34	18.4%
stage of disease at presentation	1		29	26.1%
	2		39	35.1%
	3		31	27.9%
	4		12	10.8%
Treatment	Not treated		26	13.3%
	Riluzole only		126	64.3%
	Riluzole and Edaravone		40	20.4%
	Edaravone only		4	2.0%
Presenting symptom	Fasciculations		10	4.9%
	Atrophy		10	4.9%
	Cramps		4	1.9%
	Weakness		146	71.9%
	Dysarthria		8	3.9%
	Dysphagia		15	7.3%
	more than one symptom		10	4.9%
Site of onset	Spinal		155	76.35%
	Bulbar		48	23.64%

*S*: sporadic *F*: familial, *ALSFRS-R*: Amyotrophic Lateral Sclerosis Functional Rating Scale revised; *ECAS‑EG*; Egyptian version of the Edinburgh Cognitive and Behavioral Amyotrophic Lateral Sclerosis Screen; *SD*: Standard deviation

We compared patients with spinal and bulbar onset where we found slightly older age of onset in bulbar ALS, nearly equal ALSFRS at presentation, and significantly lesser ECAS-EG median total score in bulbar patients (Table 2).

**Table 2:** Comparison between spinal ALS and bulbar ALS

	Type				Z*	*P* value
	Spinal		Bulbar			
	Median	IQR	Median	IQR		
Age	41.00	33.00–53.00	46.50	36.00–55.00	1.55	0.12
age of onset	39.00	27.00–50.50	41.00	33.00–53.00	1.44	0.15
ALS-FRS R	36.00	27.00–40.00	35.00	32.00–39.00	0.06	0.95
ECAS-EG	100.00	80.00–110.00	88.00	64.00–105.00	2.04	**0.04**

Comparison between Spinal ALS and Bulbar ALS showing older age of onset in bulbar ALS, nearly equal ALSFRS at presentation, significantly lesser ECAS-EG mean total score in bulbar patients

### Age of onset

Mean age of onset in our cohort was 39 years and IQR (28.00–51.00).

We stratified patients according to age at disease onset into three groups: juvenile “ < 25 years,” young onset “25 to 45 years,” and late onset “ > 45 years”.

### Classic ALS

Were 164 cases (81% of cases) their mean age of onset was equal to 43.6 ± 11.3

This combined **young onset** ALS cases which represented 37% of cases (76 patients) with mean age of onset equal to 35.78 ± 5.57 and **late onset** ALS cases which represented 44% of the cohort (88 cases) and had a mean age of onset 54.91 ± 5.51 (Table 3).

**Table 3:** Comparison between patients in different age groups

Age of onset in relation to type:										
			Age of onset						F*	P value
			N	Mean	SD					
Age of onset (Type)		< 25	39	18.35	5.62				558.28	** < 0.001**
		25–45	76	35.78	5.57					
		> 45	88	54.91	5.51					
*One Way ANOVA test Post hoc test showed significant difference between each two groups										
	**Age of onset**								Test value*	P value
	** < 25 y**		**25–45 y**		** > 45 y**					
	Median	IQR	Median	IQR	Median		IQR			
ALS-FRS R	35.00	26.00–40.00	34.00	26.00–40.00	36.00		27.00–39.00		0.12	0.94
ECAS-*EG* score	100.00	65.00–109.00	91.00	68.00–107.00	101.00		83.00–111.00		5.36	0.07
*Kruskal Wallis test										
					Age of onset (years)				X^2*^	P value
					** < 25**	**25–45**		** > 45**		
					%	%		%		
Sex				Male	69.2%	76.1%		67.2%	1.65	0.44
				Female	30.8%	23.9%		32.8%		
Consanguinity				No	48.8%	64.4%		78.6%	7.00	**0.03**
				Yes	51.2%	35.6%		21.4%		
Type				Sporadic	74.4%	77.3%		91.0%	6.40	**0.04**
				Familial	25.6%	22.7%		9.0%		
Positive family history of other neurological conditions				No	65.0%	66.1%		83.3%	3.69	0.16
				Yes	35.0%	33.9%		16.7%		
phenotype				Spinal	84.6%	78.9%		71.2%	2.12	0.35
				Bulbar	15.3%	21.1%		28.8%		
Extra motor affection at presentation				No	50.0%	56.4%		61.0%	0.75	0.69
				Yes	50.0%	43.6%		39.0%		
Need for nasogastric tube at presentation				No	85.3%	80.0%		78.1%	0.73	0.69
				Yes	14.7%	20.0%		21.9%		
Need for ventilation support at presentation				No	76.5%	83.5%		81.3%	0.80	0.67
				Yes	23.5%	16.5%		18.8%		
stage of disease at presentation				1	30.0%	25.0%		25.6%	8.70	0.19
				2	25.0%	26.9%		51.3%		
				3	30.0%	34.6%		17.9%		
				4	15.0%	13.5%		5.1%		
*Chi square test (FE: Fisher Exact)										

### Juvenile ALS

We found 39 JALS patients 27 males and 12 females, representing 19% of cases, 33 of them had spinal onset ALS (84%) while 6 had bulbar onset. 29 cases were sporadic (74.4%) while 10 were fALS (25.6%) which is significantly higher than other age groups (*p value 0.04*).

Comparing these age groups we found significant difference in the presence of consanguinity (*p value 0.03*); being highest in the JALS group 51% of cases (20 out of 39 cases). Moreover the presence of family history of other neurological disease was more in JALS (35%), and least in late onset ALS (16%). We also found that the presence of extra motor manifestations at presentation is highest with JALS (50% of cases) and least in late onset ALS (39%) yet insignificantly (*p value 0.69*) (Table 3).

### Rate of progression

Median ALSFRS-R score at presentation was 34.5, with IQR of (26.00–40.00). Rate of disease progression which was assessed by the mean monthly decline of ALSFRS-R was equal 0.76 ± 0.51. Comparing the rate of disease progression across age groups we found significant difference between JALS group, young onset and late onset ALS groups (*p value* < *0.001)*,with the young onset group (aged: 25–45) showing the highest rate of progression and JALS group the least rate (Table 4).

**Table 4:** a Rate of disease progression and its correlations b Relation between rate of progression and treatment categorized by age of onset

a						
	Minimum	Maximum	Mean	SD	Median	IQR
Rate of progression	-2.33	0.33	-0.76	0.51	-0.67	-1.00 – -0.67
		Rate of progression			Test value	*P* value
		Median	IQR			
Age of onset#	< 25	-0.33	-0.67 – .00		17.95*	**< 0.001**
	25–45	-1.00	-1.33 - -0.67			
	> 45	-0.67	-1.00 – -0.67			
Sex	Male	-0.67	-1.00 – -0.33		0.71**	0.48
	Female	-0.67	-1.00 – -0.67			
Site of Onset	Spinal	-0.67	-1.33 – -0.67		0.19**	0.85
	Bulbar	-0.67	-1.00 – -0.67			
Smoking	Non-smoker	-1.00	-1.33 – -0.67		0.32**	0.75
	Smoker/ex-smoker	-0.67	-1.33 – -0.67			
Type S/F	Sporadical	-0.67	-1.00 – -0.33		1.61**	0.11
	Familial	-1.00	-1.33 – -0.67			
Presenting symptom	Fasciculations	-0.67	-1.00 – -0.67		11.78*	0.07
	Atrophy	-0.67	-1.00 – -0.33			
	Cramps	-1.00	-1.33 - -0.67			
	Weakness	-1.00	-1.33 – -0.67			
	Dysarthria	-0.83	-1.00 – -0.33			
	Dysphagia	-0.83	-1.33 – -0.67			
	more than one symptom	0.00	-0.67 – 0.00			
Extra motor affection at presentation	No	-1.00	-1.33 – -0.67		3.80**	**< 0.001**
	Yes	-0.67	-1.00 – -0.50			
Need for nasogastric tube at presentation	No	-0.67	-1.00 – -0.67		2.59**	**0.01**
	Yes	-0.67	-0.83 – -0.17			
Need for ventilation support at presentation	No	-0.67	-1.00 – -0.67		3.07**	**0.002**
	Yes	-0.67	-1.00 – 0.00			
Stage of disease	1	-1.00	-1.33 – -0.67		6.67*	0.08
	2	-1.00	-1.33 – -0.67			
	3	-1.00	-1.33 – -0.67			
	4	-0.67	-1.00 – -0.50			
Cognitive impairment (ECAS < 104.5)	No	-0.67	-1.33 – -0.67		1.20**	0.23
	Yes	-0.67	-1.00 – -0.67			
Risk factors	No	-1.00	-1.33 – -0.67		9.40*	0.15
	Family History	-0.67	-1.67 – -0.67			
	Exposure to pesticides	-1.00	-1.00 – -0.67			
	Occupation	-0.67	-0.67 – -0.33			
	Smoking	-1.00	-1.33 – -0.67			
	Trauma	-1.00	-1.00 – -0.67			
	Physical activity	-0.50	-0.83 – 0.00			
Treatment	No	-0.67	-1.00 – .00		5.17*	0.16
	Riluzole	-0.67	-1.00 – -0.67			
	Riluzole and Edaravone	-0.67	-1.00 – -0.67			
	Edaravone	-0.33	-0.67 – 0.00			
b						
< 25 years:						
		Rate of progression			Test value*	*P* value
		Median	IQR			
Treatment	No	-0.17	-0.67 – 0.00		1.45	0.48
	Riluzole	-0.50	-0.67 – -0.33			
	Riluzole and Edaravone	-0.33	-1.33 – 0.00			
25–45 years						
		Rate of progression			Test value*	*P* value
		Median	IQR			
Treatment	No	-1.17	-1.33 – -0.67		4.79	0.19
	Riluzole	-1.00	-1.33 – -0.67			
	Riluzole and Edaravone	-1.00	-1.17 – -0.67			
	Edaravone	0.00	0.00 – 0.00			
> 45 years:						
		Rate of progression			Test value*	*P* value
		Median	IQR			
Treatment	No	-0.67	-0.67 – -0.67		0.64	0.89
	Riluzole	-0.67	-1.00 – -0.67			
	Riluzole and Edaravone	-0.67	-1.00 – -0.67			
	Edaravone	-0.67	-0.67 – -0.67			

a Describe the Rate of disease progression and its correlations showing significant difference with Age of onset: between group (< 25) and group (> 45). As well as, the presence of extra motor manifestations at time of presentation, Need for nasogastric tube at presentation, and Need for ventilation support at presentation

b Describe the Rate of disease progression and treatment categorized by age of onset. Showing no significant relation between treatments within different age groups

*Kruskal Wallis test

**Mann Whitney U test

#Pairwise comparison shows significant difference between (< 25) group vs. (25–45) group and between (< 25) group vs. (> 45) group

The rate of progression was uniform across the first three stages of the disease but the fourth stage showed the least progression. Yet it was statistically insignificant (*p value 0.08)* (Table 4). Moreover, the linear regression analysis for the rate of disease progression showed significant increase in disease progression in the 3rd stage compared to the 1st stage (*p value 0.02)* (Table 5).

**Table 5:** Linear regression analysis for factors affecting rate of progression

	Unstandardized Coefficients		Standardized Coefficients	Sig	95.0% Confidence Interval for B	
	B	Std. Error	Beta		Lower Bound	Upper Bound
Age of onset*						
25–45	-0.419	0.134	-0.263	**0.003**	-0.687	-0.151
> 45	-0.225	0.157	-0.127	0.155	-0.537	0.087
Male sex	0.142	0.135	0.112	0.297	-0.128	0.412
Spinal type	-0.204	0.114	-0.167	0.077	-0.431	0.022
Smoking	-0.049	0.122	-0.029	0.687	-0.293	0.194
Sporadic type	-0.035	0.107	-0.029	0.745	-0.248	0.178
Extra motor affection	0.338	0.112	0.199	**0.003**	0.116	0.560
Need for nasogastric tube	-0.172	0.184	-0.058	0.352	-0.539	0.194
Need for ventilation support	0.093	0.366	0.020	0.799	-.0635	0.822
Treatment**						
Riluzole	-0.792	0.174	-0.609	**0.000**	-1.138	-0.446
Riluzole and Edaravone	-0.730	0.192	-0.316	**0.000**	-1.113	-0.347
Edaravone	-0.373	0.383	-0.051	0.333	-1.136	0.390
Stage of disease***						
Stage 2	0.162	0.132	0.091	0.223	-0.101	0.424
Stage 3	0.396	0.175	0.192	**0.027**	0.046	0.745
Stage 4	0.550	0.306	0.151	0.077	-0.060	1.159

*Reference group: < 25 **Reference group: no treatment ***Reference group: Stage 1

The rate of progression was also correlated significantly with the presence of extra motor manifestation at presentation (*p value* < *0.001)* (Table 4).

Although it had no significant correlation with treatment intake, the linear regression analysis for the factors affecting rate of disease progression showed significant negative correlation with Riluzole therapy (*p value* < *0.0001)*.as well as combination therapy of Riluzole and Edaravone (*p value* < *0.0001)*. While there was no correlation with Edaravone only group (Table 5).

### Diagnostic delay

Median diagnostic delay was 12 month, with an IQR of (6.00–36.00). Diagnostic delay was significantly longer in younger age groups *p value* < *0.001*, it was also longer in spinal ALS (18 month) and in females (18 month), as well as patients from outside Cairo (21 month) but insignificantly (Table 6).

**Table 6:** Diagnostic delay among different groups

	Age of onset							F	*P* value
	< 25		25–45			> 45			
	Median	IQR	Median	IQR		Median	IQR		
Time from onset to diagnosis (month)	27.00	12.00–60.00	18.00	6.00–36.00		12.00	6.00–24.00	13.82**	** < 0.001**
		Time from onset to diagnosis (month)						Z*	*P* value
		Median			IQR				
Residency	Cairo	12.00			6.00–36.00			0.40	0.69
	Outside Cairo	21.00			6.00–36.00				
Gender	Male	12			6–26			1.437	0.15
	Female	18			9–30				
Site of Onset	Spinal	18.00			6.00–36.00			1.19	0.24
	Bulbar	12.00			6.00–24.00				

^*^Mann Whitney U test

^**^Kruskal Wallis test

^##^Pairwise comparison shows significant difference between group (< 25) and group (> 45)

Compares the diagnostic delay and Age of onset: Showing significant difference between group (< 25) and group (> 45). As well as, Relations between Diagnostic Delay and Residency, Gender, and Site of Onset; revealing the longer diagnostic delay in patients from outside Cairo, females, spinal onset

## Discussion

Most of the data about ALS are based on European epidemiological and genetic studies, hence, the importance of studying ALS in other regions in the world and measuring to what extent they differ from the literature.

### Clinical & demographic characteristics

In our study, there was male predominance which is in accordance with other studies [3, 4, 21]. Although male to female ratio (2.5:1) is higher than other studies [22], it is comparable to Libyan and Tunisian studies that reported a ratio of 2.3:1 and 2:1 respectively [23, 24]. Also there is a multicentre African study that reported a slightly higher ratio [25].Such male predominance could be explained by the presence of environmental factors that predispose male patients to ALS due to certain occupations that cause exposure to serious pollutants or heavy metals or require vigorous physical exertion.

Familial ALS cases were 18% of cohort, which is higher than the percentages usually reported even in the MENA region [24, 26, 27]. Notably the percentage of familial cases in our cohort was significantly higher in younger ages of disease onset; moreover the presence of family history of other neurological diseases was also higher in younger patients though insignificantly, but this points to possible genetic etiologies that are probably augmented by the presence of consanguinity in our population.

The mean age of onset in Egyptian ALS patients is 38.8 (14.4) years, which is a lot younger than the age reported in other populations; yet closer to patients from china [27], than those elsewhere [1, 22]. including studies in other Arabic and African countries like Libya Tunisia, and Morocco, that reported mean age of onset 51,55, 50 years old respectively [23, 24, 28]. as well as the multicenter study from Africa that included 8 African countries which reported mean age of onset of 53 yrs old [25], this is truly intriguing since Egypt is an Arabic north African country.

It has been postulated that age of onset is younger in less developed regions. Although the explanation is not entirely understood, it could be due to multiple factors including pollution, nutrition [27].

In our cohort, juvenile ALS patients were 19% of cases, which is a lot higher even than reported in the Tunisian population [24] Taking into consideration that both FALS and JALS are strongly linked to genetic etiologies; we attributed the higher % in our cohort mainly to the presence of consanguinity across the population. However, comparing our findings with the Tunisian population that share a lot of common social and demographic features; including consanguinity which is 34% in our study and 30% in the Tunisian study. We can conclude that beside ethnic and genetic etiologies, there are other influential environmental factors that increase or accelerate the disease process in Egypt.

Moreover, these clinical features point to regional peculiarities, specifically the genetic profile of ALS cases in the MENA region; that were proven to have a wider spectrum of genetic mutations in a recent Tunisian study [29], thus we believe regional phenotype genotype study is crucial.

The majority of our patients had spinal onset ALS (76%), which is in accordance with other studies around the world [1, 3, 24, 25]. but the percentage is higher than European studies [10, 30].

Median age of onset in bulbar ALS was 41 years with an IQR of (33–53) which is older than spinal ALS patients. This is in line with previous studies [31]. We found that spinal /bulbar onset varied across age groups showing an increased percentage of bulbar ALS with advanced age, being 16% in the juvenile group, 21% in the young onset group, and 29% in the late onset. This may explain the majority of spinal onset ALS in our cohort and justify the variation between our patients and the European studies in this point.

Moreover, the cognitive functions were more affected in bulbar ALS as denoted by significantly lesser median ECAS-EG score, which is in line with other studies [22, 32].

### Diagnostic delay

The median diagnostic delay in our study was 12 ± (6–36) months which goes with the reported in a previous Egyptian study and the literature [4, 11, 22].

We found that the diagnostic delay varied across age groups being longer with younger ages of onset, as well as in spinal onset ALS and in females; which is in agreement with a previous Egyptian study [11].

### Rate of disease progression

Showed significant difference between the age groups; denoting that age of onset is an influential prognostic factor. With the JALS having the lowest rate of progression while the young onset group (aged: 25–45) have the highest rate. Moreover the presence of extra motor manifestations at presentation was correlated significantly with the rate of progression.

The prognostic role of the site of the disease’s onset was not confirmed in our study, which is in accordance with a Chinese study [27]. We also couldn't find a correlation between rate of progression and presence of cognitive impairment which goes in line with another Chinese study [33]. We believe that these findings being common with Chinese studies which share with us the young age of disease onset could denote the presence of a different profile of prognostic factors other than those commonly reported in the literature, or at least a variable order of these factors. Which could be related to shared environmental aspects between the two populations.

### Effect of treatment

As for the treatment intake, we found no difference in the rate of progression between patients who received no treatment, those who received Riluzole only, and those with combination therapy of Riluzole and Edravone, which goes with a study that stated Riluzole doesn't slow the disease progression but only prolong the end stage [34], however it's in contrast to an Indian study showing delay in progression with therapy; that was slightly more with combination therapy [35]. Yet the regression analysis revealed significant negative correlation between disease progression and the treatment intake, with Riluzole only, and combination therapy of Riluzole and Edravone, which goes with other studies that suggested riluzole association with delayed progression. [36, 37].

We believe that, since Riluzole is claimed not to slow disease progression rates in patients at stages 2 and 3. But only prolong survival in patients at stage 4 [34], this could explain why it didn’t slow the rate of progression in our cohort, since the advanced disease stages are the hardest to maintain regular follow ups for our patients.

### Study limitation

We conducted this study at a tertiary healthcare centre which could restrain the representativeness of our data. It is an observational study which hinders the possibility of obtaining causal relationships. Moreover there is lack of data about disease outcome in so many cases; having over 40% of patients referred from all over Egypt, their follow ups get harder with disease advancement; which rendered us unable to accurately calculate the survival rate. Finally, the lack of genetic studies to identify genotype– phenotype relationships. In spite of these limitations, this study represents one of very few clinical and epidemiological studies of ALS in Egyptian patients, providing valuable information about patient's characteristics in our population.

In conclusion, the epidemiological and clinical characteristics of Egyptian ALS patients show that some aspects are in accordance with the literature. Yet, some phenotypic features differ; more familial cases, male predominance, younger age of onset, and more JALS cases; with a raised suspicion that it may not always be a benign slowly progressive form. This is notable because ALS is a fatal neurological diseases; with complicated pathophysiology, heterogeneous clinical presentations and grave prognosis. Thus, comparative regional studies are highly beneficial especially in regions where there is scarcity of information.

## Data Availability

The datasets generated during and/or analyzed during the current study are available from the corresponding author on reasonable request.
